# Empirical mode decomposition of local field potential data from optogenetic experiments

**DOI:** 10.3389/fncom.2023.1223879

**Published:** 2023-07-05

**Authors:** Sorinel A. Oprisan, Xandre Clementsmith, Tamas Tompa, Antonieta Lavin

**Affiliations:** ^1^Department of Physics and Astronomy, College of Charleston, Charleston, SC, United States; ^2^Department of Computer Science, College of Charleston, Charleston, SC, United States; ^3^Faculty of Healthcare, Department of Preventive Medicine, University of Miskolc, Miskolc, Hungary; ^4^Department of Neuroscience, Medical University of South Carolina, Charleston, SC, United States

**Keywords:** fast-spiking neurons, medial prefrontal cortex, orthogonal decomposition, cocaine, neural avalanches

## Abstract

**Introduction:**

This study investigated the effects of cocaine administration and parvalbumin-type interneuron stimulation on local field potentials (LFPs) recorded *in vivo* from the medial prefrontal cortex (mPFC) of six mice using optogenetic tools.

**Methods:**

The local network was subject to a brief 10 ms laser pulse, and the response was recorded for 2 s over 100 trials for each of the six subjects who showed stable coupling between the mPFC and the optrode. Due to the strong non-stationary and nonlinearity of the LFP, we used the adaptive, data-driven, Empirical Mode Decomposition (EMD) method to decompose the signal into orthogonal Intrinsic Mode Functions (IMFs).

**Results:**

Through trial and error, we found that seven is the optimum number of orthogonal IMFs that overlaps with known frequency bands of brain activity. We found that the Index of Orthogonality (IO) of IMF amplitudes was close to zero. The Index of Energy Conservation (IEC) for each decomposition was close to unity, as expected for orthogonal decompositions. We found that the power density distribution vs. frequency follows a power law with an average scaling exponent of ~1.4 over the entire range of IMF frequencies 2–2,000 Hz.

**Discussion:**

The scaling exponent is slightly smaller for cocaine than the control, suggesting that neural activity avalanches under cocaine have longer life spans and sizes.

## 1. Introduction

Using psycho-stimulants, such as cocaine, is a serious health problem and opens the door to neurobiological changes in limbic and cortical circuits that engage cognitive and emotive processing. Recently, we have just begun to understand the cellular adaptations that occur in the cortex following a single exposure to cocaine and their contribution to the continuous and further use of drugs of abuse (Spealman et al., 1999; Farrell et al., 2018; Goode and Maren, 2019; Reiner et al., 2019; Park et al., 2022).

The behavioral consequences of first-time cocaine use vary and appear somewhat contradictory. First-time cocaine users often report feeling a sharpening of the senses (Volkow and Swann, 1990), and anecdotal information suggests that acute cocaine increases attention. Indeed, individuals with attention-deficit/hyperactivity disorder (ADHD) will sometimes self-medicate with cocaine (Weiss and Mirin, 1986). Contrastingly, Jentsch et al. (2002) have shown that acute cocaine administration impairs performance on a reversal-learning task, and several studies have reported compromised performance during repeated acquisition tasks in monkeys (Thompson and Moerschbaecher, 1979; Evans and Wenger, 1992). Additionally, human imaging studies have shown that acute cocaine administration induces prominent prefrontal cortex activation, primarily in the dorsolateral regions (Howell et al., 2010). Furthermore, acute cocaine administration has been linked to poor impulse control (Fillmore et al., 2002; Jentsch et al., 2002; Garavan et al., 2008). Therefore, first-time cocaine use may enhance users' awareness while cognitive performance is diminished. One of the primary targets of cocaine is the prefrontal cortex (PFC; Haney et al., 2001; Fillmore et al., 2002; Garavan et al., 2008; Howell et al., 2010; Dilgen et al., 2013; Farrell et al., 2018).

In the mPFC, most of the excitatory neural population is made of pyramidal neurons (Feldman, 1984; Bannister, 2005). The main modulators of the activity of the pyramidal neural network are the calcium-binding protein parvalbumin (PV) interneurons, with PV+ fast-spiking interneurons representing the majority of these cells (Takahata and Kato, 2008; Casanova and Trippe, 2009; Kana et al., 2011). The PV+ neurons coordinate the output of the local minicolumns (Galarreta and Hestrin, 2001; Sultan et al., 2013), maintain and modulate both beta 15–30 Hz (Michael and Zoe, 2006; Hong et al., 2008; Cheng et al., 2016) and gamma 25–40 Hz (Cardin et al., 2009; Sohal et al., 2009, 2016) rhythms of the brain, and facilitate information processing (Guidotti et al., 2005; Fuchs et al., 2007; Schmidt and Mirnics, 2015). The oscillatory activity of individual neurons contributes to the observed beta and gamma rhythms of the brain (Buzsaki and Draguhn, 2004; Fujiwara-Tsukamoto and Isomura, 2008; Liddle et al., 2016). They allow task coordination (Kahana et al., 1999), support memory formation and retrieval (Roux and Uhlhaas, 2014), and signal neuropathological conditions (Orekhova et al., 2007; Peter and Wolf, 2010). The normal ongoing oscillatory activity could be reset by sensory inputs, such as visual (Kambe et al., 2015; Woelders et al., 2017) or auditory (Mercier et al., 2013) stimuli, or extrinsic stimuli, such as deep brain stimulation (Tass, 2003) or temperature (Rensing and Ruoff, 2002). Abnormal activity of PV+ interneurons has been linked to autism (Schnitzler and Gross, 2005; Levy, 2007; Orekhova et al., 2007; Peter and Wolf, 2010), schizophrenia (Lewis et al., 2005; Lewis and Hashimoto, 2007; Schmidt and Mirnics, 2015; Liddle et al., 2016), sensory hypersensitivity, and neural hyper-excitability (Gibson et al., 2008; Rotschafer and Razak, 2014; Contractor et al., 2017; Ethridge et al., 2017). Spectral analysis is a fast and suitable method for describing the statistical response of large populations of neurons.

An extensive study searching for changes in power spectral density based on subdural electrocorticographic recordings in the frequency bands 30–100 Hz somewhat arbitrarily divided the gamma band in 10 Hz increments (Crone et al., 1998). The Fourier spectral analysis did not detect any narrow-band peaks but identified distinctive power law responses in lower (30–50 Hz) compared to higher (75–100 Hz) gamma frequencies. The authors hypothesized that the neurophysiological mechanisms involving the two broad bands are significantly different (Crone et al., 1998).

It has been well-established through Fourier spectral analysis that energy distribution across different frequency bands from, e.g., subdural electrocorticographic recordings, follows a power law (Miller et al., 2009):


(1) $$\begin{array}{l} P\left(f\right)\propto Af^{-\xi }, \end{array}$$

where *P*(*f*) is the power spectral density, *A* is an amplitude factor, and ξ is the power law exponent (Miller et al., 2009), sometimes called the self-similarity parameter (Lux and Marchesi, 1999). A flat power spectral density with an exponent ξ = 0 corresponds to white noise. Pink noise is a signal whose power spectral density decreases proportionally to the inverse of the frequency, where ξ = 1. An exponent ξ = 2 is the signature of Brownian noise or a one-dimensional random walk (Milstein et al., 2009). Increasing (negative) values of the scaling exponent ξ indicate “the persistence in the time series over many different time scales” (Tolkunov et al., 2010). All power law relationships are also scale-invariant, i.e., *P*(λ*x*) = λ^ξ^*P*(*x*) for any value of the scale factor λ. Graphically, the curve describing the relationship between *x* and *y* = *P*(*x*) maintains its shape under any possible dilatation Roman and Bertolotti (2022). The power law scaling from Equation (1) is sometimes called power spectrum scale invariance because it suggests no preferred temporal or spatial scale in the signal (Shelhamer, 2007; Radulescu et al., 2012). Scale-invariant or scale-free phenomena possess the same statistical properties at any scale. Practically, the same principles or processes work across multiple temporal and spatial scales (Milne, 1998).

There is yet to be a definitive answer as to why there should be scaling laws in neural activity. One particularly appealing hypothesis links the critically self-organized systems (Bak et al., 1987) and spontaneous neural oscillations (Buzsaki, 2006). Complex systems near criticality develop correlations that decay more slowly and extend over larger temporal and spatial scales than the local scale of the underlying process (Bak, 1997). In the theory of self-organized criticality systems, pink noise for which the scaling exponent is ξ = 1 seems to be the optimal transition between order and randomness (Bedard et al., 2006). Broadband pink noise has been predicted mathematically based on a random-mood swing model and confirmed in an extensive psychiatric epidemiological survey (van der Werf et al., 2006). For example, one of the first computational models of a fully connected neural network based on non-leaky integrate-and-fire neurons showed avalanche-like activity (Eurich et al., 2002). They proved analytically and checked numerically that the avalanche sizes scale with a critical exponent of -3/2. Subsequent LFP recordings from acute slices of rat cortex showed that a power law describes the propagation of spontaneous activity in cortical networks with an exponent of -3/2 for event sizes (Beggs and Plenz, 2003). LFP recordings in cortex cultures, urethane-anesthetized rats, and awake macaque monkeys showed that, both *in vitro* and in a network model, Shannon's information capacity and information transmission are maximized at the criticality threshold (Shew et al., 2011). Therefore, the brain could operate at the criticality threshold to maximize appropriate functional criteria.

At the same time, different studies revealed a broad range of power-law relationships, some over very narrow frequency ranges and others (including ours) over a much broader frequency spectrum. An EEG study focused on the α band using wavelet analysis found power-law exponents in the range of 0.36 (eyes closed) and 0.51 (eyes open) setup, differences which were not considered statistically significant (Linkenkaer-Hansen et al., 2001). Power spectral density for neural time series obtained via functional MRI has been used to detect anxiety traits, although estimating the power-law exponents was secondary to their goal (Tolkunov et al., 2010). Large-scale realistic models mimicking the electrophysiological LFP recordings based on particular network architectures over various model neurons show Brownean noise with an exponent of ξ = 2 (Milstein et al., 2009). A recent study traced the power spectral density exponents in subcortical nuclei from the human thalamus and basal ganglia while simultaneously recording cortical activity (Bush et al., 2023). The results show an exponent of ξ = 1.3 ± 0.2 in subcortical regions compared to ξ = 3.2 ± 0.3 in the cortex (Bush et al., 2023). Although most of the EEG studies cover relatively low-frequency bands, one study, in particular, covered a wide range of frequencies 80–580 Hz and found a scaling exponent of ξ = 4.0 ± 0.1 for cortical recordings using electrocorticographic electrodes (Miller et al., 2009). The same study also found that the power spectral density scales with ξ = 2.46 ± 0.32 at frequencies lower than 80 Hz (Miller et al., 2009). One possible route to explaining the emergence of power law in neural activity could be the observation that autocorrelation of neural activity increases hierarchically across the cerebral cortex, from sensory to frontal areas (Murray et al., 2014). The broad range of power law exponents observed in experiments and modes is due to widely different targeted areas and underlying mechanisms (Beggs and Plenz, 2003). Among the most prevalent mechanism envisioned for scale invariance in neural activity is homeostatic plasticity, with different implementation details that also can drive variations in observed exponents (Friedman et al., 2012; Capek et al., 2023).

While spectral (Fourier-based) analysis provides useful quantitative analysis of power distribution across different frequency bands, it also has limitations. For example, the dynamic system must be linear, and the data should be periodic or stationary (Looney et al., 2015). For the wavelet analysis, a filter function should be selected beforehand, and one may only obtain a physically meaningful interpretation of linear phenomena (Huang et al., 1998). As the biological time series is nonlinear and non-stationary, Fourier spectral analysis and wavelet approach may give misleading results (Huang et al., 1998).

This study used a data-driven approach to modeling optogenetic data and identified the relationship between the inputs and outputs of a complex system without making any hypotheses regarding the internal processes that led to the observed output (Wang et al., 2006). Such models require a small dataset for calibration and usually have a better prediction performance over the range of tested input-output pairs (Jain and Kumar, 2007; Shrestha and Nestmann, 2009). The EMD has been proven to be an effective decomposition method for nonlinear and non-stationary data (Huang et al., 1998, 2003). Compared to wavelet and Fourier spectral analyses, the EMD better describes the local time scale instantaneous frequencies and does not need any predetermined basis functions (Huang et al., 1998; Li, 2006). With the EMD, a time series can be decomposed into a small number of orthogonal Intrinsic Mode Functions (IMFs), which are derived based on the local characteristic time scale of the data itself and describe the dynamic behavior from high-frequency to low-frequency (Huang et al., 1998, 2003; Wu and Huang, 2004; Wu, 2015). The EMD allows the analysis of biological systems on an intrinsic multi-time scale (Looney et al., 2015). The EMD was also used to investigate the neural response's time-frequency properties (Huang et al., 1998). For example, the EMD-based analysis of the oscillatory properties of spike trains in the presence of nonlinearities and non-stationarities gave better results than the traditional spectral analysis and neural network-based methods (Laurent, 1996; Averbeck et al., 2006; Bathellier et al., 2008; Alegre-Cortes et al., 2016, 2018; Wykes et al., 2016).

Optogenetics is a technique that combines optics and genetics to control and manipulate the activity of specific cells in living organisms, typically neurons in the brain. It involves genetically encoded light-sensitive proteins called opsins, which can be selectively expressed in target cells using genetic engineering techniques. The core principle of optogenetics revolves around the ability of these opsins to respond to specific wavelengths of light by either activating or inhibiting the activity of the cells in which they are expressed. For example, genetically targeted potassium channels in a rodent model of focal neocortical epilepsy showed promising therapeutic applications (Wykes et al., 2012). By precisely controlling the timing, duration, and intensity of light stimulation, neural activity can be controlled in real-time and with high spatial and temporal resolution (Dilgen et al., 2013; Sohal et al., 2016). Optogenetics has many applications, from mapping neural circuits to modulating neural activity with precise spatiotemporal control (Gholami Pourbadie and Sayyah, 2018). Optogenetics reveals the intricacies of the brain's circuitry involving neural plasticity (Eleftheriou et al., 2017) and memory (Liu et al., 2012; Ramirez et al., 2014) and helps understand the underlying mechanisms of various neurological disorders. Optogenetics has been used for manipulating and guiding cellular behavior, potentially revolutionizing tissue engineering, and regenerative therapies (Spagnuolo et al., 2020). Optogenetics techniques have been applied to treating anxiety (Allsop et al., 2014), paving the way for innovative therapeutic interventions in epilepsy (Kokaia et al., 2013; Paz et al., 2013; Peng et al., 2013; Paz and Huguenard, 2015; Wykes et al., 2016; Borges et al., 2023) and Parkinson's (Kravitz et al., 2010; Ratnadurai-Giridharan et al., 2017).

## 2. Materials and methods

### 2.1. Animal research and ethics

A detailed description of the procedures can be found in the previous papers of this series (Oprisan et al., 2015, 2018, 2019), and we only briefly summarize them here. All procedures were done following the National Institute of Health guidelines as approved by the Medical University of South Carolina Institutional Animal Care and Use Committee.

### 2.2. Experimental protocol

Male PV-Cre mice (B6; 129P2—Pval^*btm*1(*Cre*)*Arbr*/*J*^) Jackson Laboratory (Bar Harbor, ME, USA) were infected with the viral vector [AAV2/5. EF1a. DIO. hChR2(H134R)—EYFP. WPRE. hGH, Penn Vector Core, University of Pennsylvania] delivered to the mPFC as described in detail in Dilgen et al. (2013). The extracellular signals were amplified using a Grass amplifier (Grass Technologies, West Warwick, RI, USA), digitized at 10 kHz by a 1401plus data acquisition system, visualized using Spike2 software (Cambridge Electronic Design, LTD., Cambridge, UK), and stored on a PC for offline analysis. A HumBug 50/60 Hz Noise Eliminator (Quest Scientific Inc., Canada) filter canceled out the line noise. The signal was band-pass filtered by the acquisition software online between 0.1–130 kHz to obtain the LFPs. After tissue stabilization, 100 different 2s duration single-unit recordings were used to estimate the minimal laser power needed to elicit a response. For all subsequent recordings, the laser power was 25% above the above-determined minimal value (Dilgen et al., 2013). Before recording the responses to laser stimuli, LFPs were monitored for a minimum of 10min while occasionally stimulating at 40 Hz to ensure the stability of the electrode placement and the ability to induce the oscillation (Dilgen et al., 2013). Four animals were excluded from the analysis due to fluctuating levels of LFP activity (Dilgen et al., 2013).

A 473nm laser (DPSS Laser System, OEM Laser Systems Inc, East Lansing, MI, USA) delivered the light stimulation via a 1401plus digitizer and Spike2 software (Cambridge Electronic Design LTD., Cambridge, UK). The optogenetic signal is a weak biological signal easily contaminated by high-frequency noise, such as electromyographic interference, and low-frequency noise, such as baseline wander. Additional details on the experimental protocol can be found in Dilgen et al. (2013) and Oprisan et al. (2015, 2018).

### 2.3. Empirical mode decomposition analysis of nonlinear and non-stationary data

**Figure 1:**
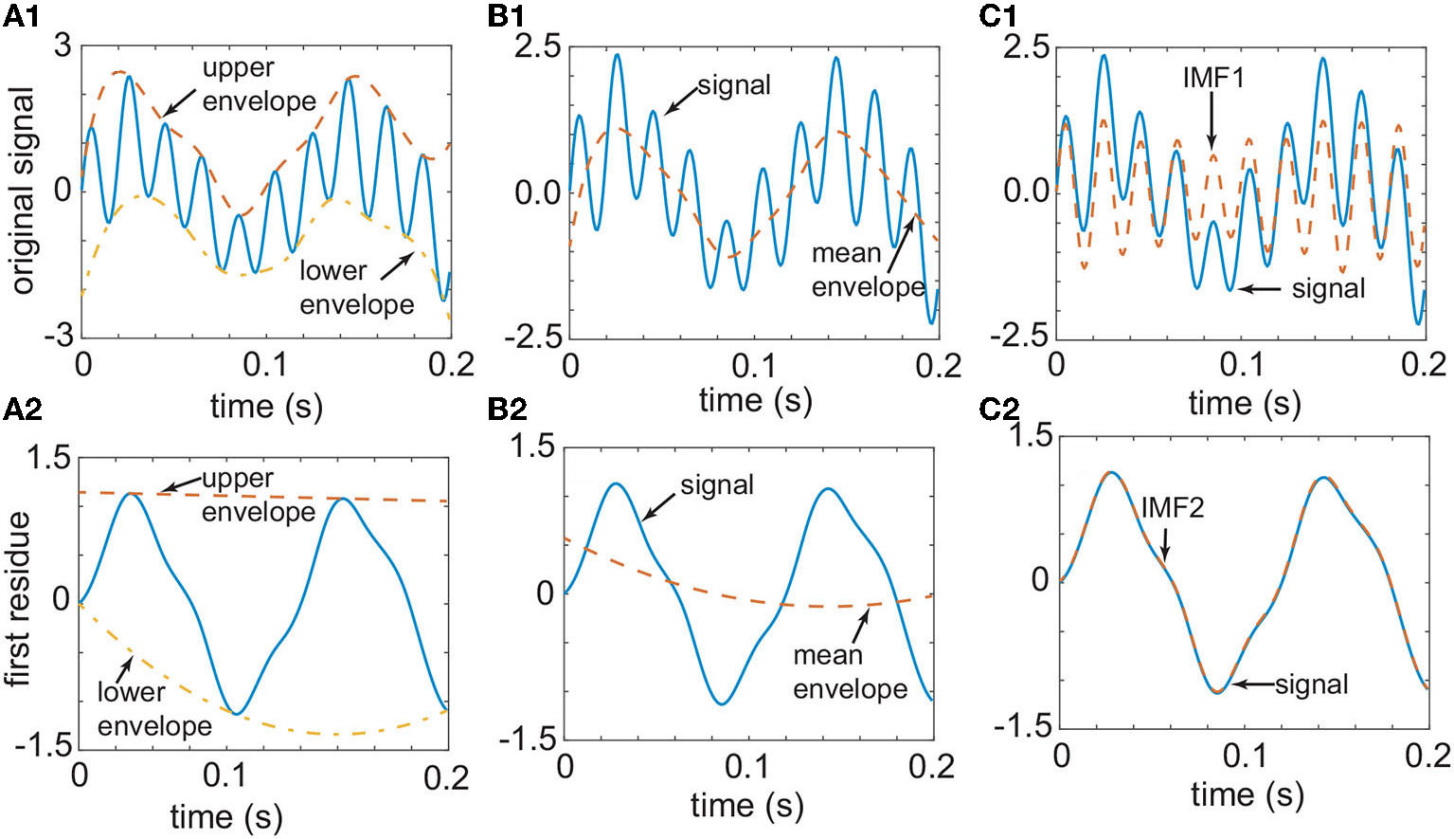
The original signal [continuous line in **(A1)**] has a smooth upper envelope [dashed line in **(A1)**] and a lower envelope [dashed-dotted line in **(A1)**]. The two envelopes from **(A1)** determine the mean envelope in **(B1)** (dashed line). By subtracting the mean envelope of B1 from the signal, one obtains the first IMF in **(C1)** (dashed line). The difference between the signal [continuous line in **(C1)**] and the first IMF [dashed line in **(C1)**] gives the first residue in **(A2)** (continuous line). The process depicted in **(A1–C1)** is repeated with the signal shown in **(A2)** until one obtains the second IMF in **(C2)**. This one-dimensional signal was generated from an analytic function to demonstrate the use of the EMD technique.

The EMD decomposes nonlinear and non-stationary signals into oscillatory components using Hilbert-Huang transform (Huang et al., 1998; Zhu et al., 2013). Unlike Fourier-based time series analysis, EMD makes no *a priori* assumption for underlying time series structures. Therefore, the EMD is suitable for analyzing time series consisting of multiple periodic components, e.g., climatic data or biomedical signals. The EMD is self-adaptive and based on the local characteristic time scale of the data. The EMD has been highly efficient in processing non-linear, non-stationary, and time-varying data in neuroscience (Lang et al., 2010). IMFs are a series of data sequences with different eigenscales. Each IMF function has the same number of extrema and zero crossings, with its envelopes being symmetric about zero (Huang et al., 1999). The iterative process of extracting IMFs is known as sifting and consists of the following steps:

1. identification of the local extrema of the signal *x*(*t*);
2. interpolation of the maximal and minimal points set to obtain an upper envelope, *x*_*u*_(*t*), and a lower envelop, *x*_*l*_(*t*), respectively (see the dashed lines in Figure 1A1 labeled “upper envelope” and “lower envelope,” respectively);
3. compute the average of the two envelopes *m*(*t*) = [*x*_*u*_(*t*)+*x*_*l*_(*t*)]/2 (see the dashed lines in Figure 1B1 labeled “mean envelope”);
4. subtraction of the average *m*(*t*) from the original signal to get *d*(*t*) = *x*(*t*)−*m*(*t*), and repeat steps (1-4) until *d*(*t*) satisfies the two conditions for being an IMF (see the dashed lines in Figure 1C1 labeled “IMF1”; Rilling et al., 2003).

The two conditions for correct IMF definition are (1) the number of extrema and the number of zero-crossing must be either equal or differ by one at most, and (2) at any point, the mean value defined by the envelope of the local maxima and the envelope of the local minima is zero (Huang et al., 1998). Once the first IMF1 is generated, the residual signal *r*(*t*) = *x*(*t*)−*IMF*1(*t*) is regarded as the original signal, and steps (1–4) above are repeated to yield the second IMF (see the dashed lines in Figure 1A2 labeled “upper envelope” and “lower envelope,” respectively; see the dashed lines in Figure 1B2 marked “mean envelope,” and the dashed lines in Figure 1C2 labeled “IMF2”), and so on.

The iterations stop when the amplitude of the residue falls below a pre-determined small value so that further sifting would not yield any valuable components (Huang et al., 1998). The stopping criterion impacts the orthogonality of the decomposition and energy leakage between IMFs (Huang et al., 1998; Echeverria et al., 2001; Flandrin et al., 2004; Attoh-Okine et al., 2008; Fele-Zorz et al., 2008). The stopping criterion guarantees the computation of a finite number of IMFs. The original signal *x*(*t*) can be expressed as:


$$x\left(t\right)=\underset{j}{\sum }c_{j}\left(t\right)+r\left(t\right),$$

with


$$c_{j}\left(t\right)=Re\left(a_{j}\left(t\right)e^{i\phi \left(t\right)}\right)=Re\left\{a_{j}\left(t\right)\right\}exp\left(i\overset{t}{\underset{-\infty }{\int }}\omega_{j}\left(t^{\prime }\right)dt^{\prime }\right),$$

where *c*_*j*_(*t*) represents the IMFs and *r*(*t*) the remaining non-oscillating trend or residual. The plots of the amplitude, *a*_*j*_(*t*), and phase, ϕ(*t*), vs. time for each IMF represents a Hilbert-Huang spectrogram (not shown; Attoh-Okine et al., 2008). Although IMFs are empirically determined, they remain orthogonal to each other and may therefore contain independent physical meaning (Wu et al., 2007; Lo et al., 2009; Yang et al., 2011). The EMD decomposes different time series into IMFs, each oscillating at specific time scales. The EMD also de-trends the time series to produce a zero-mean distribution, i.e., removes the residual component *r*(*t*) from raw data. Therefore, the EMD reduces spurious regression and multi-collinearity in subsequent multiple linear regression analyses (Yang et al., 2011; Masselot et al., 2018).

#### 2.3.1. IMF orthogonality

Although the IMFs are orthogonal, the Hilbert-Huang transform may produce other than truly orthogonal decompositions in practice. For example, if some high-frequency components of IMF1 leak into the second IMF2, the EMD suffers from a mode mixing problem determined by the widely disparate scales of a single IMF (Huang et al., 1998). Furthermore, if the waveforms between two IMFs are similar, the decomposition suffers from another mode mixing problem in that a signal resides in different IMF components (Huang et al., 1998). To quantify the degree of orthogonality and estimate the extent of mode mixing, we compute the Index of Orthogonality (IO) and the Index of Energy Conservation (IEC; Chen et al., 2006; Ho and Hung, 2022). If any two IMFs, such as *c*_*i*_(*t*) and *c*_*j*_(*t*), are orthogonal, then IO is zero, whereas if there is a total overlap between them, the IO is close to unity. The IO is a normalized crosscorrelation between IMFs and is defined as follows:


(2) $$\begin{array}{l} IO=\frac{\underset{t}{\sum }\overset{N+1}{\underset{j=1,i\neq j}{\sum }}c_{i}\left(t\right)c_{j}\left(t\right)}{\underset{t}{\sum }x^{2}\left(t\right)}, \end{array}$$

where the residual *r*(*t*) is treated as the (*N*+1)-th IMF. Other studies do not exclude autocorrelation from IO computation, i.e., they do not require *i*≠*j* in Equation (2) (Shen et al., 2021). In that case, the diagonal elements of the IO matrix are not zero, like in our case, but proportional to the fraction of the signal's energy in that particular IMF mode. Here the energy of an IMF mode *c*_*i*_(*t*) is defined as the temporal summation (or integration) of the amplitudes squared, i.e., $\underset{t}{\sum }c_{j}^{2}\left(t\right)$.

The IEC is the normalized sum of energy (integral of the square of the amplitudes; Chen et al., 2006; Ho and Hung, 2022):


(3) $$\begin{array}{l} IEC=\frac{\underset{t}{\sum }\overset{N}{\underset{i=1}{\sum }}c_{i}^{2}\left(t\right)}{\underset{t}{\sum }{\left(x\left(t\right)-r\left(t\right)\right)}^{2}}. \end{array}$$

The IEC is the sum of energy in all IMFs normalized by the original signal *x*(*t*) minus the trend given by the residual *r*(*t*). If the IEC is close to unity, then the decomposition is close to lossless. If IEC is not comparable to unity, the issue may be the stopping criteria (Chen et al., 2006).

#### 2.3.2. IMF energy vs. frequency

Most neuroscience-related studies have concentrated on narrow-band power-law scaling of power density distribution vs. frequency (Crone et al., 1998; Feige et al., 2005; Milstein et al., 2009; Tolkunov et al., 2010; Bush et al., 2023). Wu and Huang (2004) conducted an extensive theoretical study of the effect of noise on broadband energy vs. frequency spectrum using EMD. We investigated the possible power law relationship between IMF energies and the instantaneous frequency by plotting them on a log-log scale. If Equation (1) holds, then


log(P(f))∝-ξlog(f)+logA,

where the slope of the log-log plot determines the scaling exponent ξ. An often neglected requirement of power-law scaling is that such relationships are defined only in the limit of an infinite system (Henkel et al., 2008). In our EMD of LFPs, the frequency range covered three orders of magnitude, which is higher or at least at par with the most detailed multielectrode-based recordings (Beggs and Plenz, 2003).

## 3. Results

The spectra of instantaneous bandwidths and IMF frequencies are adaptive to the nature of data. It is convenient to map the instantaneous frequency bands of the EMD decomposition into EEG frequency bands, such as δ band: 0.5–3.5 Hz; θ band: 3.5–8 Hz, α band: 8–13 Hz; β band: 13–30 Hz; and γ band: 32–80 Hz (Tatum, 2014).

### 3.1. EMD of mPFC optogenetic response to a brief laser pulse

**Figure 2:**
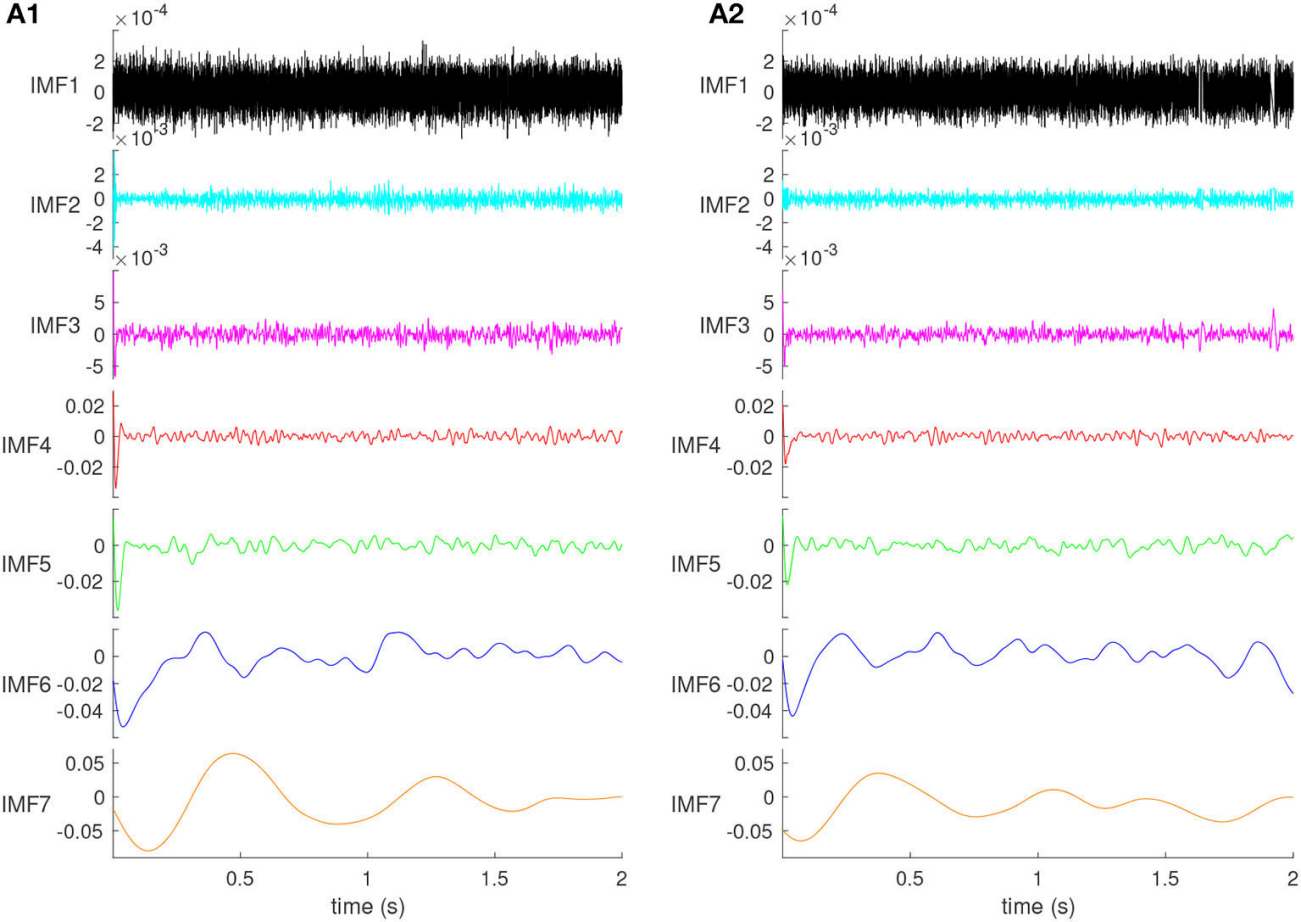
The IMFs of the control **(A1)** and cocaine-injected mice **(A2)**. The amplitude of the IMFs increases with their order. The first two IMFs contain mostly high-frequency noise. Their time scale is faster than 10 ms laser pulse duration, and they do not produce a visible impulse response. The effect of the brief laser pulse on the network's activity is visible at the beginning of all IMF3 through IMF7.

**Figure 3:**
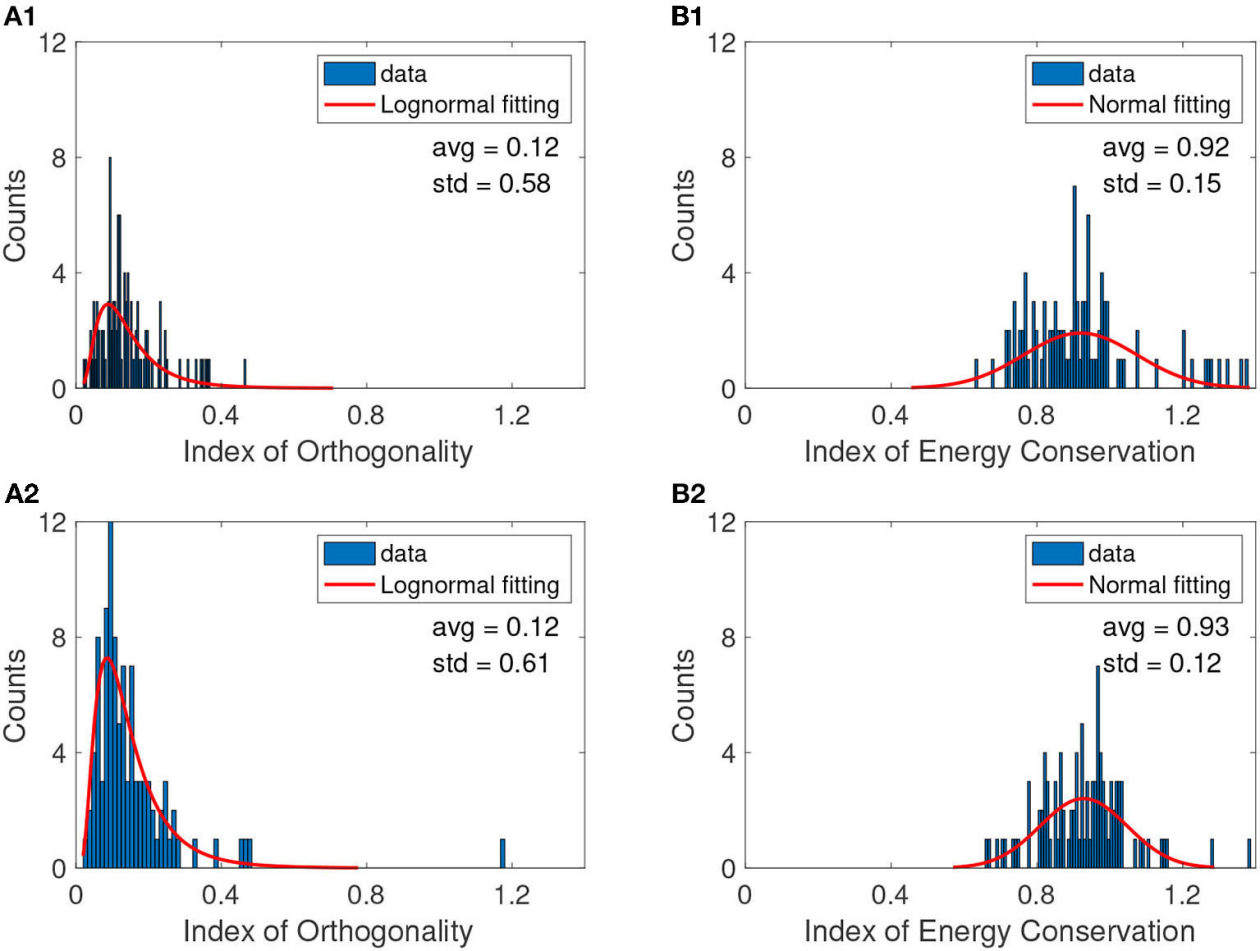
A typical example of IO distributions before **(A1)** and after cocaine **(A2)**. The continuous red line is the smooth lognormal fit with the logarithmic averages (avg) and standard deviations (std) shown on each panel. The IEC distributions before **(B1)** and after cocaine **(B2)** are best fitted with a Gaussian with a continuous red line. The null hypothesis regarding the distribution was checked with a 99% confidence level in all cases. Table 1 shows the logarithmic mean values *avg*_*log*_ for lognormal fit. The figure panels show the linear mean obtained as *avg*_*lin*_ = exp(*avg*_*log*_).

Through trial and error, we found that decomposing the LFP into seven IMFs produces one IMF in the γ band: 32–80 Hz (see Figures 3A1, B1), which is relevant for the cognitive functions of the mPFC. The 2s recordings were decomposed into seven orthogonal IMFs (see Figure 2), and one residual (not shown), capturing different frequency bands of the original LFP signal, some overlapping with the EEG frequency bands. The IMFs plotted in Figure 2 are the average IMFs over the 100 trials for the same representative animal before (Figure 2A1) and after (Figure 2A2) cocaine injection, respectively. All figures, except (Figure 8) summary, are for the same animal out of the six used in this experiment.

The amplitudes increase with IMF's order (see Figure 2). Moreover, the IMF amplitudes for control (Figure 2A1) and cocaine-injected mice (Figure 2A2) of the same order are generally in the same range. Figures 2A1, A2 show that the first two orders, IMF1 and IMF2, are composed mainly of high-frequency noise. This noise should be removed from the original signal. Because the main component of the second-order IMF2 is still high-frequency noise, the noise and signal have not been separated well, and the first type of mode mixing problem has occurred. The optic coupling with the neural network is strong, as revealed by the significant dips at the beginning of IMF3 through IMF7.

### 3.2. Orthogonality of EMD decomposition and mode mixing

While theoretically, the EMD generates orthogonal IMFs, in practice, orthogonality may not be achieved due to noise or numerical errors. We computed the distribution of the IO (Chen et al., 2006; Ho and Hung, 2022) according to Equation (2) over 100 trials for the same animal before (Figure 3A1) and after cocaine (Figure 3A2). In every case, the null hypothesis that the lognormal distribution is the best fit for IO was verified at a 99% confidence level. We used a Kolmogorov-Smirnov test to verify that the selected distribution is a good fit (Massey, 1951; Chakravarti et al., 1967; Marsaglia et al., 2003; Hill and Lewicki, 2005; Steinskog et al., 2007).

Here, the probability density function for lognormal distribution was


$$f\left(x\right)=y_{0}+\frac{1}{\sqrt{2\pi }x\sigma }\exp\frac{-{\left(ln\left(x\right)-\mu \right)}^{2}}{2\sigma^{2}},$$

where *y*_0_ is the offset, μ is the mean of the logarithmic values, and σ is the standard deviation of the logarithmic values. From the lognormal distribution of the IO, we found that average values before and after cocaine are similar, *avg*_*log*_≈−2.09, corresponding to *avg*_*lin*_ = exp(*avg*_*log*_)≈0.12 on a linear scale. The overall IO computed above (see Figure 3 and Table 1) offers an average estimate of orthogonality of all IMFs for each trial before Figure 3A1 and after cocaine Figure 3A2. While the IO is not zero as we would expect for an ideal EMD decomposition, the mean average over all conditions and mice is 0.13, which aligns with the findings of other studies for low values of sifting iterations (Molla et al., 2006; Ponomaryov et al., 2021).

**Table 1:** Summary of mean and standard deviations of lognormal distributions for the Index of Orthogonality (IO) and the normal distribution for the Index of Energy Conservation (IEC).

**bc**	**Mean** ±**Std IO**	**Mean** ±**Std IEC**	**ac**	**Mean** ±**Std IO**	**Mean** ±**Std IEC**
# 1	−2.09 ± 0.58	0.92 ± 0.15	# 1	−2.09 ± 0.61	0.93 ± 0.12
# 2	−1.99 ± 0.53	0.93 ± 0.19	# 2	−2.37 ± 0.64	0.95 ± 0.17
# 3	−1.94 ± 0.55	0.90 ± 0.13	# 3	−1.95 ± 0.54	0.98 ± 0.16
# 4	−1.84 ± 0.53	0.90 ± 0.13	# 4	−1.95 ± 0.42	0.91 ± 0.15
# 5	−2.00 ± 0.66	0.95 ± 0.11	# 5	−2.08 ± 0.56	0.95 ± 0.16
# 6	−2.25 ± 0.53	0.95 ± 0.11	# 6	−2.39 ± 0.62	0.95 ± 0.10

We also verified with a 99% confidence level the null hypothesis that the normal distribution best fits IEC. Representative examples are shown in Figure 3B1 for trials before cocaine (bc) and in Figure 3B2 after cocaine (ac) injection. We used a *t*-test to compare the means of the IO bc group vs. IO ac, and similarly for IEC means (Krause, 2011; Beath and Jones, 2018). For the data in Table 1, the *t*-test for IO bc vs. IO ac showed the data come from statistically identical groups at a 99% confidence level with a *p*-value of 0.3304. The IEC data are also statistically identical at a 99% confidence level with a *p*-value of 0.1633.

While the IEC is not exactly equal to unity, as in the case of ideal decompositions, its average of 0.94 ± 0.14 also aligns with similar studies that find such values acceptable for noisy data (Ho and Hung, 2022).

**Figure 4:**
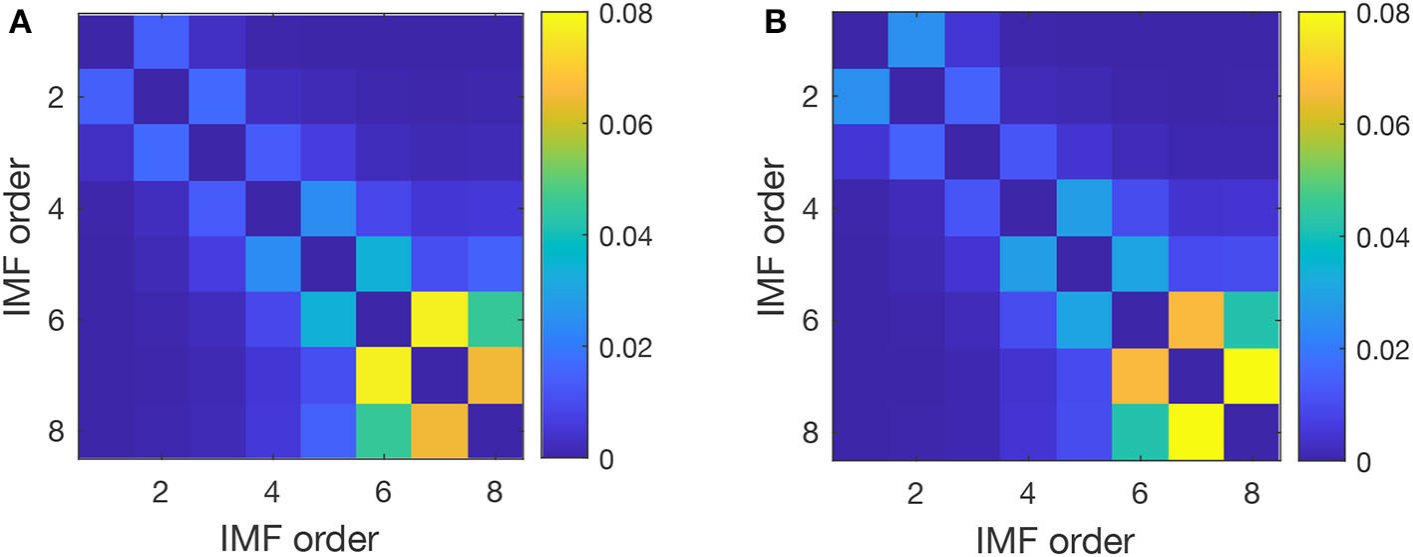
Pairwise index of orthogonality averaged over all trials for a typical subject before **(A)** and after cocaine **(B)**. The crosscorrelation of the IO decreases with the distance from the diagonal, which suggests that only successive IMFs experience some degree of mode mixing. The most significant IO is between IMF 6, with a mean frequency of 5 Hz (θ band of EEG is 3.5–8 Hz), and IMF 7, with a mean frequency of 2 Hz (δ band of EEG is 0.5–3.5 Hz).

Furthermore, we estimated the pairwise IO for every two IMFs averaged across all trials to detect possible mode mixing where the IO deviates significantly from zero. The average values of IO crosscorrelation for pairs of IMFs shown in Figure 4 have similar patterns for control (before cocaine in Figure 4A) and after cocaine (Figure 4B). We considered all seven IMFs and the residual in calculating the pairwise IO. Ideally, we expect zero crosscorrelation between IMFs. Some degree of crosscorrelation exists between any successive IMFs, as seen from the first line parallel to the diagonal in Figure 3. The only significant jump in IO crosscorrelations, to 0.078 for control and 0.066 for cocaine, occurs between IMF6 and IMF7. However, even in this case, the mode mixing is negligible as the IO values are below the 95% significance level for a mode mixing (Chen et al., 2006; Molla et al., 2006; Ho and Hung, 2022).

### 3.3. Energy-frequency analysis of IMFs

**Figure 5:**
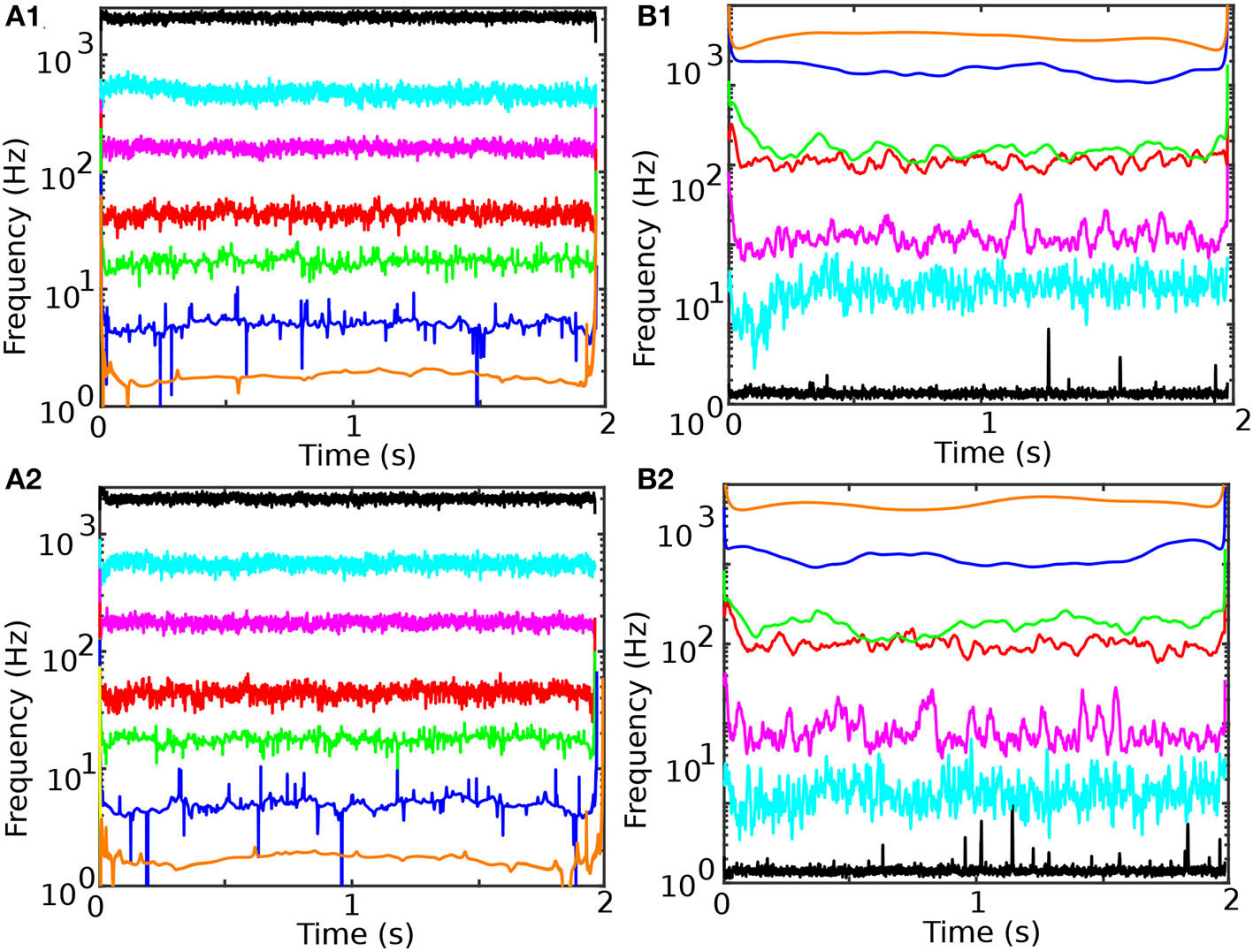
Temporal evolution of instantaneous frequency and energy content of the seven IMFs for control **(A1, B1)** and cocaine-injected mice **(A2, B2)**. The first two IMFs have instantaneous frequencies of over 2,000 and 500 Hz, respectively. Such high-frequency IMFs in the LFPs decomposition are noise. IMF3 mean is in the range of 170 Hz, IMF4 in the range of 40 Hz (γ band of EEG is 32–80 Hz), IMF5 in the range of 20 Hz (β band of EEG is 13–30 Hz), IMF6 in the range of 5 Hz (θ band of EEG is 3.5–8 Hz), IMF7 in the range of 2 Hz (δ band of EEG is 0.5–3.5 Hz). The energy of IMFs (the square of the IMF amplitude) increases with the IMF order **(B1, B2)**. The last data point in all panes suffers from the EMD edge effect.

The instantaneous frequencies in Figure 5A1 (before cocaine) and Figure 5A2 (after cocaine) were obtained by computing the first derivative of the instantaneous phase of the Hilbert-Huang transform. While the average instantaneous phase monotonically increases over time, it has an occasional negative slope due to noise, which leads to unphysical negative values for its derivative, i.e., the instantaneous Hilbert-Huang frequency. Among many solutions for overcoming the negative instantaneous frequencies due to noise (Huang et al., 2009), we adopted phase smoothing over ten samples, i.e., over 1 ms. We tested a wide range of smoothing durations, and ten samples is a reasonable compromise between removing the negative frequencies and still being small enough to give ten smoothed data points over the 10 ms stimulus duration.

The energies shown in Figure 5B1 (before cocaine) and Figure 5B2 (after cocaine) were computed as the square of absolute values of the smoothed IMF amplitudes shown in Figure 2. While the frequency bands are relatively well-separated by the individual IMFs (Figures 5A1, A2), the IMF4 and IMF5 have some degree of overlapping (see Figures 5B1, B2).

**Figure 6:**
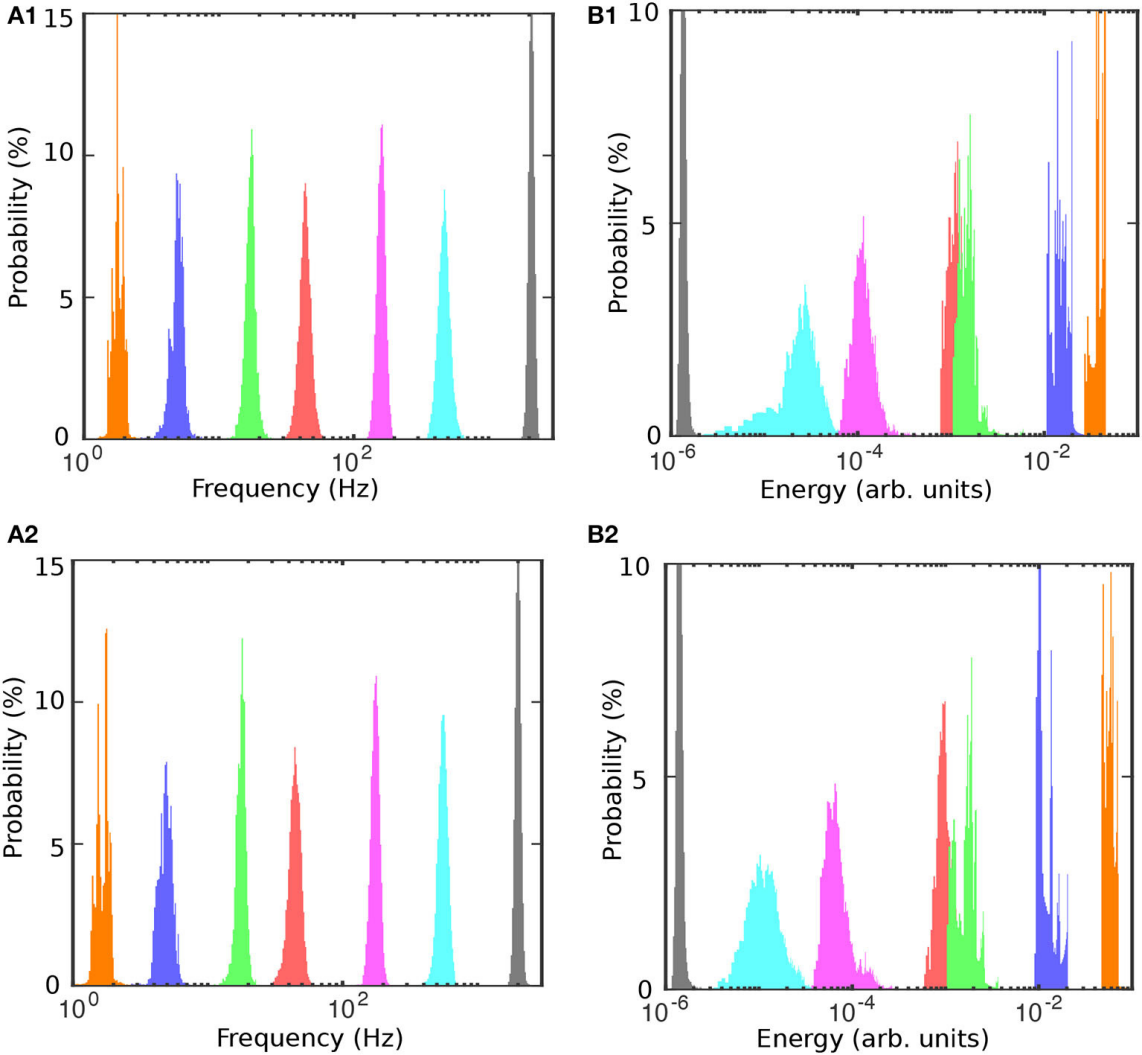
Frequency and energy histograms before **(A1, B1)** and after cocaine **(A2, B2)**. The frequencies are well-separated and normally distributed around the mean [**(A1)** before cocaine and **(A2)** after cocaine]. The energy distributions for IMF4-IMF5 and IMF6-IMF7 pairs slightly overlap.

To determine the mean and standard deviation of the instantaneous frequency spectrum for each IMF, we first tested the normality of frequency distributions (see Figure 6A1 before and Figure 6A2 after cocaine). The distribution of frequencies around the mean values of the corresponding IMFs is well-fitted with Gaussian curves with a 99% confidence level, and a summary is presented in Table 2. We also found that energy distributions are Gaussian with slight skewness (see Figures 6B1, B2). While frequency distributions around the mean values of each IMF (see Figures 6A1, A2) do not overlap, some overlap occurs in the energy distributions of IMF4 and IMF5 (see Figures 6B1, B2). The IEC also captured the overlap.

**Table 2:** Mean and standard deviations of frequencies for all IMFs before cocaine (bc) and after cocaine (ac) injection for the same animal used for Figures 2–7.

**Condition**	**IMF1**	**IMF2**	**IMF3**	**IMF4**	**IMF5**	**IMF6**	**IMF7**
Bc	2, 080 ± 96	476 ± 50	161 ± 12	44 ± 5	17 ± 3	5 ± 1	1.9 ± 0.8
Ac	2, 010 ± 97	559 ± 47	176 ± 13	45 ± 5	18 ± 2	5 ± 1	1.7 ± 0.8

Based on Table 2, we found that both the control (before cocaine) and after cocaine injection frequency scales of IMFs follow exponential decay, i.e., *frequency*∝*e*^−*n*/τ^, where *n* = 1…7 is the IMF's index. The e-fold exponents are τ = 0.78 ± 0.02 for the control and τ = 0.82 ± 0.02 for the cocaine case. Given that the e-fold exponents τ are statistically identical within the standard deviation, the “natural” frequency scales embedded in the signals are identical. As a result, a direct comparison of IMFs for control vs. cocaine results is possible without additional corrections.

**Figure 7:**
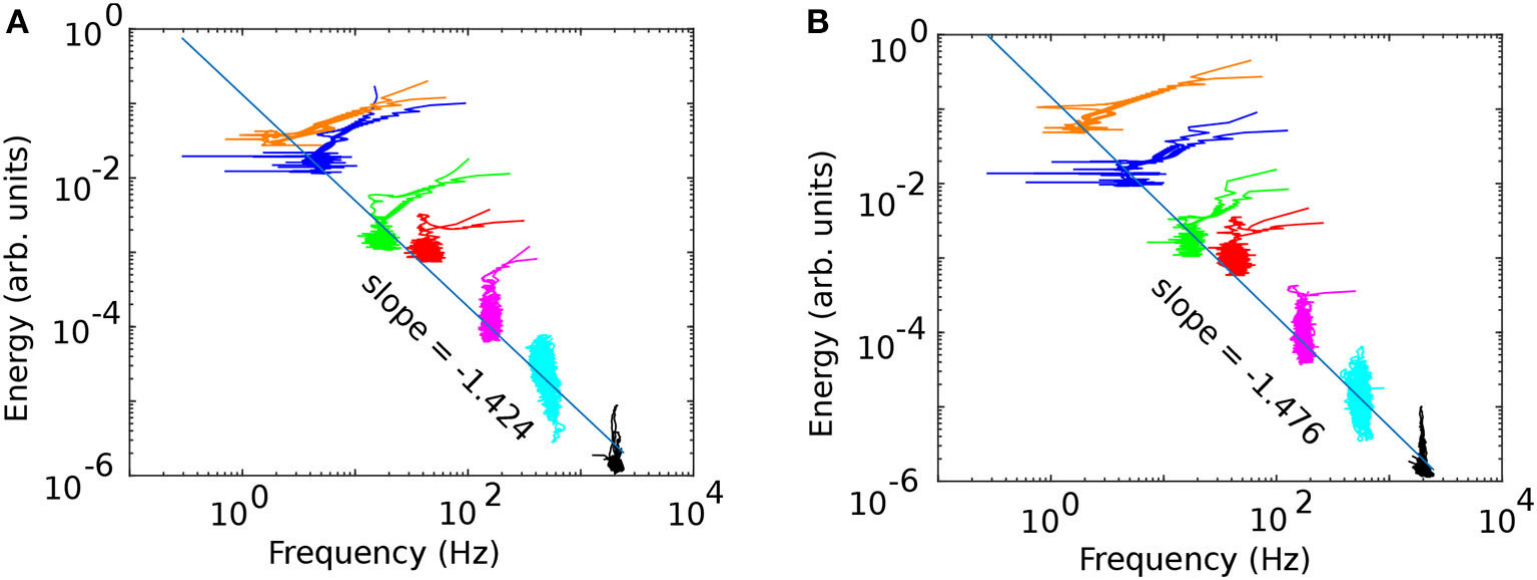
The frequency vs. energy diagrams [**(A)** before and **(B)** after cocaine] identify the seven separate clusters extracted from the original LFPs. The log-log plots show good liner fits, which indicates a power laws relationship between the energy and frequency, i.e., *energy*∝*frequency*^−ξ^.

The energy vs. frequency plots in Figure 7A (before cocaine) and Figure 7B (after cocaine) show seven clusters corresponding to the identified IMFs. Figure 7 is for the same animal out of six as in the previous figures. We also tested the EMD decomposition of the data with fewer IMFs and found that they led to a significant overlap of clusters in energy vs. frequency plots (not shown). Power laws emerge from the energy vs. frequency plot, where the power law exponents equal the straight line slopes shown in Figure 7 log-log plots.

The slopes and intercepts for the log-log plots of all six animals are shown in Table 3, together with the adjusted *R*^2^ values.

**Table 3:** The slope and the intercept of the log-log plot of energy vs. frequency show relatively stable values across all six animals both before cocaine (bc) and after cocaine (ac).

**bc**	**Slope** ±**Std**	**Intercept** ±**Std**	*R* ^2^	**ac**	**Slope** ±**Std**	**Intercept** ±**Std**	*R* ^2^
# 1	−1.424 ± 0.001	−0.882 ± 0.001	0.978	# 1	−1.476 ± 0.001	−0.836 ± 0.001	0.986
# 2	−1.405 ± 0.001	−0.859 ± 0.001	0.97	# 2	−1.253 ± 0.001	−1.34 ± 0.01	0.995
# 3	−1.624 ± 0.001	−0.826 ± 0.001	0.951	# 3	−1.352 ± 0.001	−1.16 ± 0.03	0.855
# 4	−1.349 ± 0.001	−0.778 ± 0.002	0.95	# 4	−1.236 ± 0.001	−1.11 ± 0.02	0.904
# 5	−1.314 ± 0.001	−0.856 ± 0.001	0.901	# 5	−1.28 ± 0.01	−1.08 ± 0.02	0.898
# 6	−1.2 ± 0.1	−1.565 ± 0.002	0.934	# 6	−1.38 ± 0.01	−1.708 ± 0.001	0.901

The adjusted *R*^2^ coefficient indicates a good data fit. A representative linear fit from one animal is shown in Figure 7.

**Figure 8:**
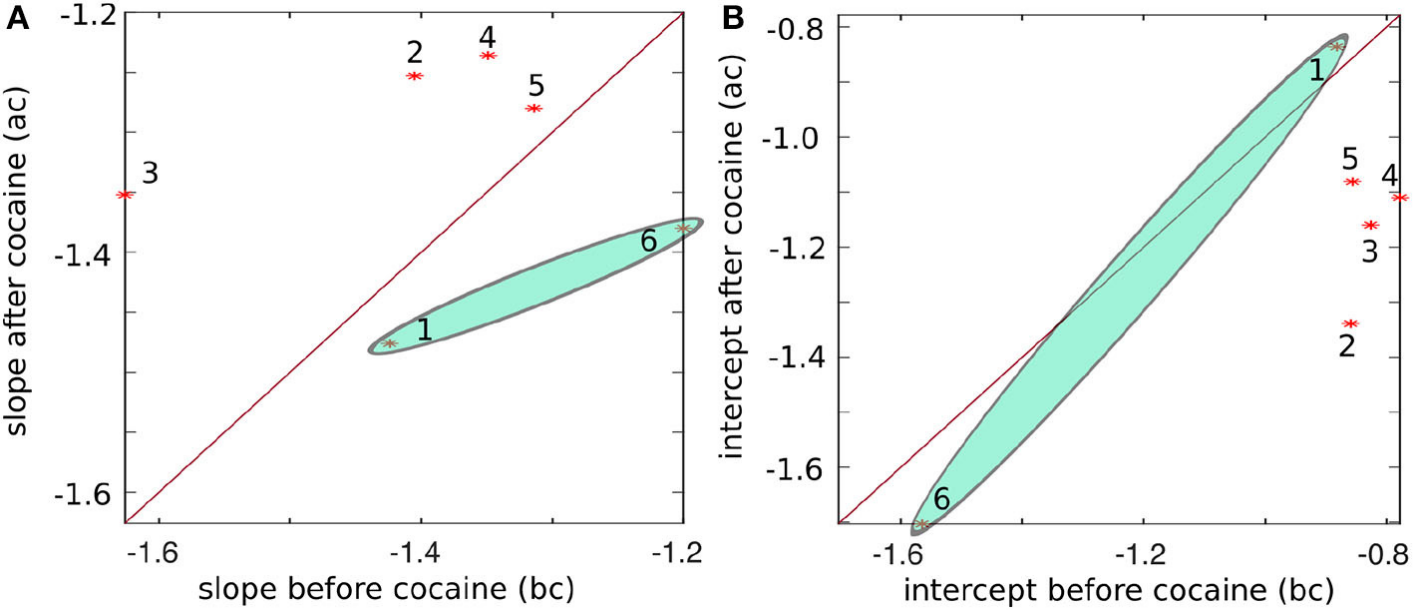
The slope of the log-log linear fit of energy vs. frequency before vs. after cocaine **(A)** and the intercept before vs. after cocaine **(B)** for all six animals (marked with stars and numbered). The continuous line corresponds to equal values before and after cocaine, i.e., the two conditions are indistinguishable along this line. Except for subjects #1 and #6, the power law exponents (the slope of the log-log plot) have a systematically smaller negative exponent after cocaine. Similarly, the intercepts have the smallest negative values before cocaine.

The plot of the slopes in Figure 8A and intercepts in Figure 8B of the log-log linear fit of energy vs. frequency summarized in Table 3 suggests some systematic patterns. Our null hypothesis, represented by the continuous line in Figure 8, is that there is no difference between the values before and after cocaine. We performed a K-means clustering analysis and found two data clusters in the power law exponents shown in Figure 8A. Subjects # 1 and # 6 fall below the separation line in Figure 8A and form the first data cluster, while the other four subjects are above it, i.e., the power law exponent after cocaine is slightly smaller (in absolute value) after cocaine. We know from a previous study using the same animals that the data for animal # 1 represents an outlier, as seen in Figure 7 of Oprisan et al. (2018). In the study mentioned above, we found that the estimated time of zero crossing of the autocorrelation function after cocaine was significantly longer compared to before cocaine. However, data from all five other animals in the same study showed that the correlation time in cocaine cases was significantly shorter than the corresponding control values. This study also confirms that data from animal # 1 continues to represent an outlier when comparing the slopes and intercepts of the log-log plot of energy vs. frequency fit (see Figure 8). This study's EMD data also suggests that animal # 6 could be another outlier, at least in light of Figure 8 (see the shaded ellipses).

## 4. Discussion

The brain is a complex system with interconnected subnetworks. Neural activity avalanches initiated, for example, by sensory inputs, can die out if the network has sparse connectivity and the neurons do not have enough dendritic processes to reach a minimal sensitivity threshold that propagates the initial avalanche (Moretti and Munoz, 2013). On the other hand, a too-tightly connected network could instantly propagate any neural activity avalanche across the entire brain, such as those propagated during epileptic seizures. It has long been speculated that the brain maintains a fine balance between chaotic neural activity and total silence by constantly evolving, adapting synaptic strengths, pruning connectivities, and tuning their sensitivity curve (Shew et al., 2011). Criticality means a system operates at the boundary between microscopic and macroscopic dynamics where neural activity avalanches driven by local interactions and microscopic details can lead to large-scale macroscopic events that trigger activity synchronization across entire networks. In critically self-organized systems, such large-scale events triggered by microscopic local activity have size distributions that obey power-laws (Levy and Solomon, 1996). At criticality, long-range correlations occur among distant elements of the system, which make small local fluctuations or minute sensory inputs to neural systems reach global effects over a wide range of temporal and spatial scales. Such behavior, described by power laws, is called scale independence (or invariance; Goldenfeld, 1992). Power laws in critically self-organized systems extend past the size distribution of neural activity avalanches found from *in vitro* (Beggs and Plenz, 2003; Mazzoni et al., 2007; Pasquale et al., 2008) and *in vivo* (Petermann et al., 2009; Hahn et al., 2010; Capek et al., 2023; Salners et al., 2023) experiments. Such power laws include different macroscopically measurable quantities, such as the duration distribution of functional connections in EEG recordings (Lee et al., 2010), the duration of neural avalanches (Ehsani and Jost, 2023), and the power spectrum. While most studies found that a power-law exponent of neural avalanche duration is around −1.5 (Beggs and Plenz, 2003; Millman et al., 2010; Cowan et al., 2013; Hesse and Gross, 2014), steeper exponents were induced by dopamine modulation (Stewart and Plenz, 2006), and by D1 receptor antagonists (Gireesh and Plenz, 2008). For example, D1 receptors set the sensitivity threshold for neural avalanches (Stewart and Plenz, 2006) and strengthen the coupling between pyramidal and fast-spiking PV+ neurons (Seamans and Yang, 2004). The pyramidal-PV+ cells network is known to set the gamma rhythm (Bartos et al., 2007). In general, steeper exponents reduce the occurrence of large avalanches and spatial correlations (Stewart and Plenz, 2006).

As we notice from Figure 7, the energy and frequency of IMFs scale as *energy*∝*frequency*^−ξ^ where the power law exponent ξ is determined by the slope of the log-log plot, which is about −1.39 ± 0.14 before cocaine and −1.39 ± 0.09 after cocaine. Based on the average values obtained from Table 3, there is no statistical difference between the power law exponents before and after cocaine. At the same time, the plot of the slope (Figure 8A) and the intercept (Figure 8A) of log-log fits of energy vs. frequency indicate two distinct clusters of data, one of which contains animal #1 (a known outlier from a previous study; Oprisan et al., 2018, 2019) and animal # 6. While the data for animal # 6 could be a new outlier that only the EMD reveals (as opposed to the zero crossing of autocorrelation used in the previous study), a definitive answer would require further investigation. Our previous study (Oprisan et al., 2019) did not detect that # 6 is also a potential outlier because the autocorrelation method used for estimating the time lag for delay embedding in that study only estimated linear correlations among data. The EMD is a more powerful tool that can handle non-stationary and nonlinear data, which could be why we can now better discern data clustering. Suppose both data sets from #1 and # 6 are outliers. In that case, the EMD allows us to distinguish between cocaine and control conditions based on the power law exponent of energy dependence on frequency.

Assuming both data from animals #1 and # 6 are outliers (based on our K-means clustering), the power law exponents differ slightly for the four data sets that cluster together. For example, the power law exponent before cocaine would be −1.42 ± 0.14 and after cocaine −1.28 ± 0.05. Reimann et al. (2013) conducted an extensive modeling study addressing the connection between the power laws exponents observed in LFP experiments and the underlying cellular mechanisms responsible for such results. They simulated the LFP using a reconstructed, multi-compartmental model of the rodent neocortical column that included dendritic and somatic compartments with voltage- and ion-dependent currents, realistic connectivity, and probabilistic AMPA, NMDA, and GABA synapse (Reimann et al., 2013). They found that below 40 Hz, the active membranes gave a scaling exponent ξ = 1.0 ± 0.2, whereas for passive membranes ξ = 0.9 ± 0.1. For the frequency range 40–1,000 Hz, they found ξ = 2.0 ± 0.4 and ξ = 3.7 ± 0.1, respectively. They concluded that “spiking and spike-related currents contribute to low LFP bandwidths traditionally considered to reflect purely synaptic activity” (Reimann et al., 2013).

Experiments using bipolar high-impedance microelectrodes (Destexhe et al., 1999) within a cat's parietal association cortex gave LFPs with good spatial localization of the signal. They allowed an accurate comparison of local vs. global power-law exponents of neuronal activities (Bedard et al., 2006). The reconstructed synaptic currents (modeled by simple exponential relaxation processes) based on experimentally recorded spikes showed a scaling exponent ξ = 2. The synaptic currents model could not explain the experimentally estimated scaling exponents of ξ = 1 for frequencies below 20 Hz and ξ = 3 for the frequency range 20–65 Hz. They concluded that the complex structure of the extracellular media that combines current flows in the conductive fluids and capacitive effects due to the high density of membranes produces the ξ = 1 scaling (Bedard et al., 2006).

The wide range of power law exponents found in different brain areas and frequency ranges could also be determined by the multifractal nature of brain organization. It is desirable to start from first principles and build neural ensembles that fit the observed dynamics and then characterize their evolution using statistical tools, such as power law dependence of energy distribution across frequency bands. This is an old argument raised almost a century ago by Einstein, who considered that probabilities must follow dynamics, not vice versa. In other words, the power spectral density vs. frequency should be derived from the equations describing the individual neurons and their connectivities. The ubiquity and simplicity of power law relationships revealed across brain regions might be deceptive compared to the complexity of neurons and their synaptic connections (Beggs and Plenz, 2003; Friedman et al., 2012; Capek et al., 2023). This is why theoretical foundations and underlying mechanisms must be worked out first to make testable predictions. Otherwise, “there is the danger for this field to become adrift in a sea of empiricism devoid of theory and with little explanatory power and generality” (Marquet et al., 2005). Besides multifractality, another reason we observe multiple scaling laws with different exponents in different brain areas could be due to the finite-size scaling limit, i.e., we are not measuring the true scaling exponents because they are only defined for a truly infinite system.

## 5. Conclusion

Oscillatory activity is ubiquitous in the neural circuitry of the brain. Assigning frequency components in LFPs to different EEG bands can provide information regarding pathological spectral power distribution. The EMD can identify different IMFs of neural activity while providing an effective noise reduction and trend elimination method. Noise is usually associated with the highest frequencies in the EMD decomposition and trends with the lowest frequencies or residuals. De-trending time series is necessary for correct frequency band separation. The unknown trends are generally described by analytic equations with parameters estimated by the least square fitting or maximum likelihood methods. However, LFP data usually show nonlinear characteristics that are difficult to estimate, which leads to inaccurate de-trending results. The EMD can identify and separate the trends for any time series without prior assumptions because the EMD is adaptive, regardless of the nonlinear and non-stationary nature of the data. As a result, EMD makes frequency scale identification more accurate and reliable.

We found that the LFPs recorded from the mPFC during the optogenetic experiment have orthogonal decompositions following statistically different patterns than the white noise. In our experiments, the IMF energy scales with the instantaneous frequency obtained from Hilbert-Huang transform as *energy*∝*f*^−ξ^ with an average power law exponent ξ≈1.4. While our study on mPFC of mice is the only one we could find that covers a very broad frequency range 2–2,000 Hz, our results support the criticality hypothesis and previously reported critical exponents for avalanche size of ξ = 1.5 in awake rhesus monkeys (Petermann et al., 2009), acute mPFC slices of adult rats (Stewart and Plenz, 2006), mature organotypic cultures and acute slices of rat cortex (Beggs and Plenz, 2003), superficial cortex of awake mice (Capek et al., 2023), *in vivo* and *in vitro* rat cortical layer 2/3 (Gireesh and Plenz, 2008), adult cats under anesthesia (Hahn et al., 2010), dissociated cortical neurons from rat embryos cultured onto micro-electrode arrays (Pasquale et al., 2008), and in conductance-based computational models (Ehsani and Jost, 2023).

We also found a slightly smaller power law exponent of ξ = 1.28 ± 0.05 for cocaine than ξ = 1.42 ± 0.14 for control. Given that a steeper exponent reduces the likelihood of observing larger neural avalanches and spatial correlations (Stewart and Plenz, 2006), a smaller power law exponent for cocaine suggests potentially larger and longer-lasting neural avalanches. One likely reason for the differences, as suggested by a detailed biophysical model, is the active membrane currents that adjust in the presence of cocaine rather than synaptic currents (Reimann et al., 2013).

A limitation of the current study is the mode mixing present among IMFs, also reflected in the IO and IEC values. A common approach toward mode de-mixing we will explore in the future is by using the ensemble EMD technique (Schlotthauer et al., 2009; Wu and Huang, 2009; Wu et al., 2009), which is counterintuitive since it adds controlled noise to the data to enhance the signal-to-noise ratio. More recently, EMD was enhanced using a weighted sliding window over the dataset (Zeiler et al., 2012) and local integral mean (Ponomaryov et al., 2021).

While our findings suggest that the local neural network changes the critical exponent of neural activity under cocaine, more detailed studies are necessary. We are exploring multi-electrode optrodes that would allow us to map network connectivities through crosscorrelation among single electrode critical exponents computed as described in this study. By combining the electrophysiology we carried out in this study with simultaneous calcium fluorescence imaging, future studies could check the consistency of neural avalanche critical exponents across different modalities.

## Data availability statement

The raw data supporting the conclusions of this article will be made available by the authors, without undue reservation.

## Ethics statement

All procedures were done in accordance with the National Institute of Health guidelines as approved by the Medical University of South Carolina Institutional Animal Care and Use Committee.

## Author contributions

SO designed the computational approach, performed EMD data analysis, and wrote the manuscript. XC contributed to data analysis and reviewed the manuscript. TT conducted the experiment, collected the data, and reviewed the manuscript. AL designed the experiment and reviewed the manuscript. All authors contributed to the article and approved the submitted version.
